# Impact of Feed Bunk Space on the Prevalence of Torsion and Foreleg Distal Asymmetry in Holstein Cows

**DOI:** 10.3390/ani14202930

**Published:** 2024-10-11

**Authors:** Luis Jesús Barrera-Flores, Rafael Rodríguez-Martínez, Francisco Gerardo Véliz-Deras, Guadalupe Calderón-Leyva, Viridiana Contreras-Villarreal, Ulises Noel Gutiérrez-Guzmán, Jorge Arturo Bustamante-Andrade, Amaury Esquivel-Romo, Robert Hagevoort, Martín Alfredo Legarreta-González

**Affiliations:** 1 Programa de Doctorado en Producción Agropecuaria, Universidad Autónoma Agraria Antonio, Narro Unidad Laguna, Torreón 27054, Mexico; luis.barrera@ujed.mx; 2 Facultad de Agricultura y Zootecnia, Universidad Juárez del Estado de Durango, Venecia 35100, Mexico; ulisesnoelg@yahoo.com.mx (U.N.G.-G.); jorge.bustamante@ujed.mx (J.A.B.-A.); amauryer@ujed.mx (A.E.-R.); 3 Departamento de Ciencias Médico Veterinarias, Universidad Autónoma Agraria Antonio Narro, Torreón 27054, Mexico; velizderas@gmail.com (F.G.V.-D.); gpecalderon06@gmail.com (G.C.-L.); dra.viridianac@gmail.com (V.C.-V.); 4 Agricultural Science Center at Clovis, New Mexico State University, Clovis, NM 88101, USA; dairydoc@nmsu.edu; 5 Universidad Tecnológica de la Tarahumara, Chihuahua 33180, Mexico; mlegarre@uttarahumara.edu.mx; 6 Posgraduate Department, Fatima Campus, University of Makeni (UniMak), Makeni City 00232, Sierra Leone

**Keywords:** animal welfare, cow locomotion, foreleg torsion, distal asymmetry, feed bunk design, principal component analysis

## Abstract

**Simple Summary:**

The prevalence of foreleg distal asymmetry and its relationship with the size of the feed bunk were studied in two farms (G60 and G100) in northern Mexico. The prevalence was 27.6% (G60: 31.4%, G100: 22.4%); principal component analysis revealed that the weight variables associated with leg diseases were the farm (G60) and three out of twelve pens in G100. Furthermore, the analysis indicated that the weight variables for leg diseases were the farm (G60) and three out of twelve pens in G100. The prevalence was related to the width of the feed bunks and the width of the manure crust on them. The proper cleaning of feed bunks and their adequate design and construction are strategies that can reduce lameness and premature culling of cows.

**Abstract:**

Leg torsion and distal asymmetry (LTDA) among cows reared on intensive farms in the Comarca Lagunera region of northern Mexico may be indicative of underlying health concerns. To ascertain whether the incidence of LTDA is associated with trough measurements and with productive, reproductive, and disease variables, the prevalence of LTDA was determined in lactating dairy cows. The data were derived from two intensive dairy farms in northern Mexico (G60: 2043 cows in 13 pens with 142.0 m of linear feed bunk space and 65.0 m of pen depth; G100: 2227 cows in 12 pens with 215.0 m of linear feed bunk space and 49.0 m of pen depth). The cows were observed over the course of a week to identify any macroscopic lesions indicative of LTDA. Cows exhibiting lesions were assigned a value of “1”, while those without lesions were assigned a value of “0”. Furthermore, data regarding other limb diseases (LDs) were collected and subjected to analysis. A comparison was conducted to ascertain the impact of reproductive, productive, and feed bunk size variables on the prevalence of LTDA and other LDs. To ascertain the prevalence of LTDA and LDs, a chi-squared test was employed. The prevalence of LTDA was found to be 27.6% (G60: 31.4%, G100: 22.4%). No association was identified between LTDA and the variables included in the study. However, a correlation was identified between LDs and the following variables: total width, distance from the edge, width from the feed bunk to the tramp, and the presence of a manure crust on the walls. Principal component analysis (PCA) was conducted to examine the correlation between LDs and various factors at the farm and pen levels. The findings indicated that the distance from the feed bunk to the trough, the presence of manure crusts on the walls, and the breeding time were associated with LDs in G100, as well as in three of the twelve pens (302, 306, and 308) within G100. The findings suggest that the prevalence of LDs is associated with an increase in the width of the feed bunk and the width of the manure crust on the walls, particularly in pregnant cows. The results permit the conclusion that LTDA and LDs are associated with the measurements of the feed bunks, the absence of manure cleaning of the feed bunks, and gestation. This association gives rise to significant health issues for Holstein cows on these farms, with more than one in four cows affected. To reduce the prevalence of LTDA in lactating dairy cows, it is recommended that the standard measurements for feed bunk design be adhered to. This will reduce the prevalence of LTDA and LDs, promote the cleaning protocols to avoid the accumulation of manure crusts, and facilitate close monitoring of pregnant cows, thereby alleviating the impacts of these foot pathologies on animal welfare.

## 1. Introduction

**Figure 1:**
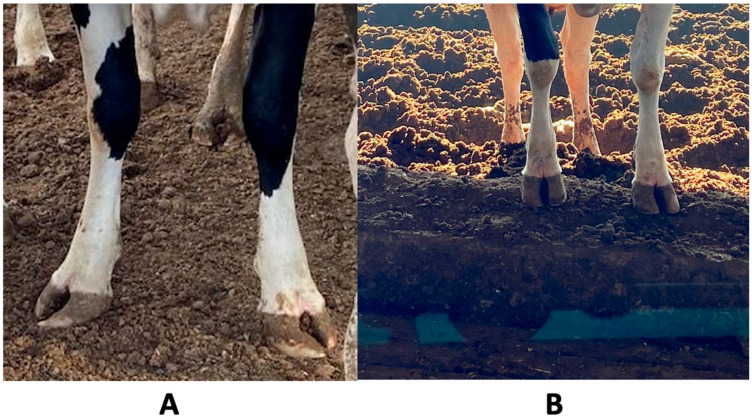
(**A**) Positive cow exhibiting limb torsion and distal asymmetry (LTDA), and (**B**) negative cow. In the positive cow, the position of the open anterior legs and the larger size of the internal toes of both extremities are observed, which are absent in the negative cow.

Lameness in dairy cows has a significant adverse effect on both animal welfare and production [1]. Furthermore, lameness can have a deleterious impact on cow welfare by impeding their ability to move without discomfort [2]. The prevalence of lameness in cattle in modern dairy production systems is high [3,4]. Cows have been observed to exhibit a preference for walking on soft surfaces [5,6]. Moreover, the prevalence of injuries to cows’ feet decrease when cows walk on grass or sandy soil [5,7]. One of the primary predisposing factors for lameness in cows is leg torsion and distal asymmetry (LTDA) [8]; this can be defined as the presence of a macroscopic lesion that manifests as torsion and growth of the hoof wall in the internal frontal phalanx, which is far from the fetlock joint, which causes inflammation and locomotion problems in dairy cattle (Figure 1). Similarly, the phenotype of the high-producing Holstein Friesian breed, with its large animals where adult females can weigh up to 680 kg, is regarded as a contributory factor in the appearance of lameness in intensive production systems [9,10,11].

In numerous contemporary dairy farm housing systems, cows are afforded only limited access to soft soil, which represents a notable deficiency in their overall care. Notwithstanding the regular functional hoof trimming, which serves to reduce the length discrepancy between the hooves and to mitigate the overload of the front and rear lateral hooves, the prevalence of sole ulcers and other foot disorders remains considerable. It seems prudent, therefore, to provide dairy cows with pens with soft soil and pastures, especially during peripartum, when the hooves are particularly susceptible to the disease [12]). This suggests that dairy cattle have not successfully adapted to manufactured intensive housing systems, where concrete is employed as the facility’s floor material. One of the primary predisposing factors for cow lameness is LTDA [8]. However, in the majority of research studies, forelimb asymmetry has been the primary focus, while torsion has only been investigated in a limited number of cases [8,13,14,15].

The prevalence of lameness is higher in intensive livestock farms [16,17], as is the case on farms in the Comarca Lagunera area in north-central Mexico. The region is home to a dairy cow population of 506,217, representing 20.33% of the national herd and producing 2,730,752 thousand liters of milk per year, which constitutes 21.75% of the country’s total [18]. The objective of the study was to evaluate the prevalence of lameness in dairy cows on large dairy farms in the Comarca Lagunera area and to analyze the variables that cause this pathology and their association with productive, reproductive, and disease variables in this important dairy area. This study assessed a greater number of animals than in previous studies, with the objective of identifying the primary cause of LTDA and thus preventing its adverse effects on animal welfare [19].

## 2. Materials and Methods

### 2.1. Overview

All experimental and animal handling procedures were conducted following the guidelines for the ethical use, care, and welfare of research animals at the international [20] and national levels [21] and with institutional approval (UAAAN-UL-18-3059).

### 2.2. Location, Weather Conditions

The study was conducted on two large dairy farms in north-central Mexico (25°52′39″ LN, 103°23′39″ LO), a region characterized by a dry climate with an average annual temperature of 21 °C, ranging from 0 °C (winter) to 37 °C (summer), with rainfall occurring from June to September with an annual average of 266 mm [22].

### 2.3. Description of the Experimental Area

The pens utilized in the study were constructed with concrete cow alleys measuring 15 cm in thickness. The alleys were reinforced with a 20 × 20 cm electro-welded rebar mesh exhibiting a concrete strength of 400 kg/cm^2^ with 1.27 cm aggregate. The alleys were 3.5 m in width and sloped laterally at a rate of 0.7%, a design feature that facilitates mechanical cleaning. The surface of the pen was composed of a mixture of clay-rich soil and fine sand. The sidewalk floors in corrals were equipped with eight channels, each measuring 2.5 cm in width and depth, and designed to provide a non-slip surface. The head gates were constructed from galvanized stanchions, measuring 3.05 m in length, which were mounted on schedule 80 steel support posts. The aforementioned gates were inserted into the feed curb and were of similar concrete strength to that of the walkways. The gates were equipped with a self-locking mechanism and five holes. All soil surfaces, all the sidewalk floors in corrals, and all the head gates were similar in all the pens.

**Figure 2:**
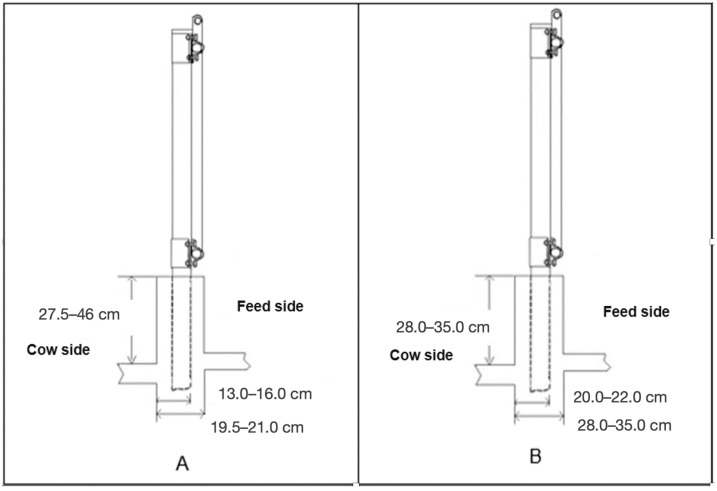
Section of feeder wall of the pens of farm G-60 (**A**) and the pens of farm G100 (**B**).

The first farm (G60) was home to 2043 lactating Holstein cows, which were evaluated in 13 pens. The pens were 142 m in length (along the feed lane) and 65 m in depth, with a slope of 1.5%. The height of the inner wall of the feed bunker exhibited a range of 27.5–46 cm, while the width of the trap support wall at the troughs varied from 19.5 to 21 cm. Furthermore, the distance from the inner edge of the trap support wall to the inner base of the traps exhibited variability, with measurements ranging from 13 to 16 cm (Figure 2A). A total of 2227 lactating Holstein cows in 12 pens were evaluated on the second farm (G100). The pens on this farm were 215 m in length (along the feed lane) and 49 m in depth, with a slope of 1.5%. The height of the inner wall of the feed bunker exhibited a range of 28 to 35 cm, while the width of the trap support wall at the feeders demonstrated a fluctuation of 28.5–37 cm. Furthermore, the distance from the inner edge of the trap support wall to the inner base of the traps varied between 20 and 22 cm (Figure 2B).

### 2.4. Characteristics of the Animals

A total of 4270 lactating Holstein Friesian cows were evaluated on both farms. Cows in G60 were from one to six lactations, while in G100 they were from two to five lactations. The cows in both dairies were milked three times per day and had been in milk for less than 120 days. A fourth milking was conducted at the peak of the lactation period. The cows in both dairies had unlimited access to drinking water. The diet provided to the cows was based on their nutritional requirements [23] and consisted of the following components: corn silage, alfalfa hay, whole cottonseed, soybean meal, canola meal, rolled corn, molasses, a mineral package, a mycotoxin binder, and sodium bicarbonate.

### 2.5. Determining the Prevalence of LTDA

To determine the prevalence of LTDA, the front legs of all cows were observed for a seven-day period between the hours of 5:00 a.m. and 3:00 p.m. while they were either ingesting feed or resting, without disturbing their rest. Cows exhibiting twisting and growth of the hoof wall at the anterior distal internal phalanx were classified as positive for LTDA and assigned a value of “1”. Cows that did not exhibit any lesions were considered negative for LTDA and assigned a value of “0”. Moreover, photographic documentation of the cows in question was obtained. Photographs were taken with a cell phone camera (iPhone 11, Apple Inc., Zhengzhou, China; 12-pixel resolution) at a distance of 1.0 m, at an approximate height of 1.20 m, and at an angle of 30–40°. This approach permitted a more comprehensive examination of the lesions. Furthermore, data from additional LDs were documented.

### 2.6. Analysis of the Effect of Variables on the LTDA and LD Presence

The objective was to relate the presence of LTDA and LDs with the physical characteristics of the feed bunk on both farms and with productive, reproductive, and disease variables. To this end, the dimensions of the feed areas were measured: the width of the feeder walls (WF), the distance from the edge of the wall to the trap (WTD), and the height of the feed bunk walls (HF). Furthermore, the width (SWF) and height (SHF) of the manure crust adhering to the wall were also measured. All these measurements are expressed in cm. The resistance of the manure crust to manual pressure was also evaluated, with scores of 1, 2, and 3 assigned to indicate the crust’s softness (1), medium softness (2), and hardness (3), respectively. Following standard practice, the measurements were taken with a 6 m retractable measuring tape (Trupper FCG-6M). The productive variables of interest include dairy farm (DF), corral number (GN), lactation (LC), days in milk (DIM), last production 24 h (PLD), body condition score (BCS), ruminant movement average 24 h (RA; Cow Watch, NEDAP Agri, Netherlands), and activity average 24 h (AA; Cow Watch, NEDAP Agri, Netherlands). The reproductive variables were as follows: the breeding number (IA); the status (ST, calving = 1, abort = 2, empty = 3, heat = 4, inseminated = 5; pregnant test I = 6, pregnant test II = 7, dry = 8); the pregnancy time (BT); the open days (OD); the calving interval (CI). The data presented herein were obtained from farm systems (DP21, Westfalia Surge, Oelde, Westfalia, Germany). The variables designated as “diseases” were limb torsion and distal asymmetry (LTDA) and leg diseases (LDs: crooked, foot rot, and lameness).

### 2.7. Statistical Analysis

The distribution of LTDA and LD cases was evaluated using the chi-square test and a principal component analysis (PCA) was employed to ascertain the variables associated with the prevalence of both of them. The *X*^2^ and PCA analyses were conducted using the R programming language [24]. Only those differences that reached a level of statistical significance (*p* ≤ 0.05) were accepted for further consideration.

## 3. Results

The present study considered cows belonging to the G60 and G100 groups, with a minimum of one and maximum of six lactations. In order to analyze the level of interaction between variables of production, reproduction, as well as variables of pen infrastructure and cleaning, and their level of causality for LTDA and LDs, several variables were considered.

### 3.1. Statistical Descriptive of Numerical Variables of Study

The statistical descriptive of the variables analyzed in this study is presented in Table 1, where mean, standard deviation, minimum, and maximum values for each one the variables can be observed.

### 3.2. Principal Component Analysis

#### 3.2.1. Asymmetry/Torsion Prevalence (LTDA, LDs)

The 14 selected variables were subjected to an analysis grouped by LTDA and LDs using the principal component analysis (PCA) method. The results indicated that a total inertia was explained by the first two components, amounting to 43.5%. Nevertheless, no distinction could be made between the positive and negative groups of cows. Table 2 presents the results from the *Χ*^2^ analyses from the variables related with LTDA, which highlights the differences between negative and positive groups (*p* < 0.05) with a percentage of 72.4 for negative and 27.6 for positive cows. In relation to the status of cows, we also found differences (*p* < 0.05 for this variable, with the biggest value for inseminated cows (41.8%) and the lowest value for aborted cows (0.00)). Finally, leg disease variables showed differences (*p* < 0.05), with lameness being the most important associated variable (94%).

With regard to the prevalence of LTDA and LDs (Table 3) in each of the study barns, both were observed to be higher (*p* < 0.05) in G60 than in G100 (LTDA, G60 = 31.4% vs. G100 = 22.4%; LDs, G60 = 17.6% vs. G100 = 1.7%). Significant differences were observed between the two farms for LTDA and LDs in the proportion of positive cows (LTDA: G60 (0.31), G100 (0.22) [*χ*^2^ (1, 41.196), *p* < 0.001]; LDs: G60 (0.18), G 100 (0.02) [*χ*^2^ (1, 268.01), *p* < 0.001].

#### 3.2.2. Principal Component Analysis: LTDA

To analyze LTDA with the variables of the study, these were grouped according to their association resulting in a total of 43.5% variation explained by the first two components (PC1, 22.6% and PC2, 20.9%); Table 4.

**Figure 3:**
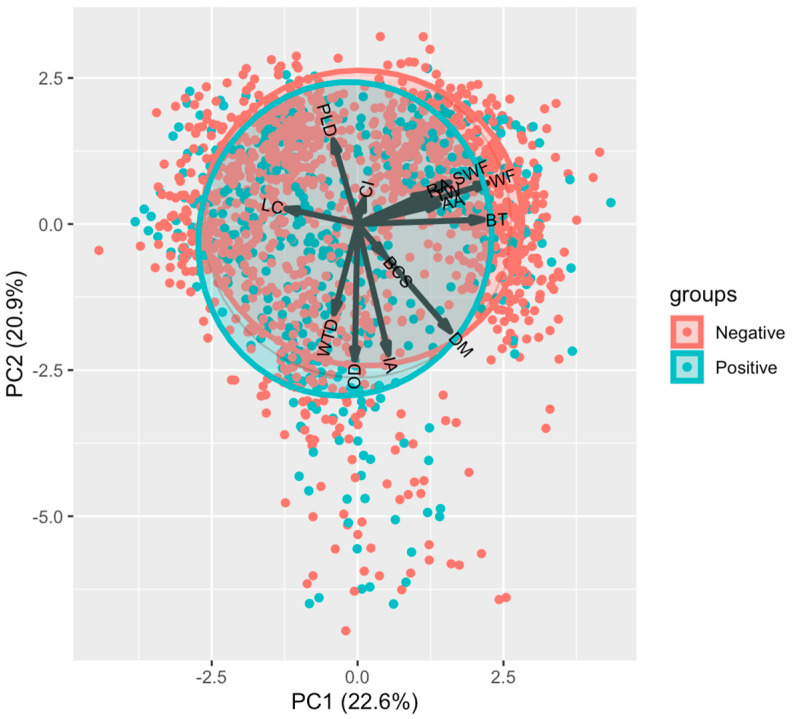
Principal component analysis for foreleg torsion and distal asymmetry (LTDA) in cows from two farms in northern Mexico. PC1 = Principal component 1; PC2 = Principal component 2, DM = Days in milk, PLD = Last production in 24 h, IA = Breeding days, OD = Open days CI = Calving interval, BCS = Body condition score, BT = Pregnancy time, RA = Ruminant movement average last 24 h, AA = Activity Average in last 24 h, WF = Distance from the edge of the wall to the trap, WTD = Height of the feed crust, and SWF = Height of manure crust adhered to the wall.

PCA found no association between the variables included in the study and LTDA (Figure 3), although it is observed that the variables SW, SWF, and BT are grouped in the same direction.

#### 3.2.3. Principal Component Analysis: LDs

To analyze LDs with the variables of the study, these were grouped according to their association resulting in a total of 32.8% variation explained by the first two components (PC1, 26.0% and PC2, 16.8%); Table 5.

**Figure 4:**
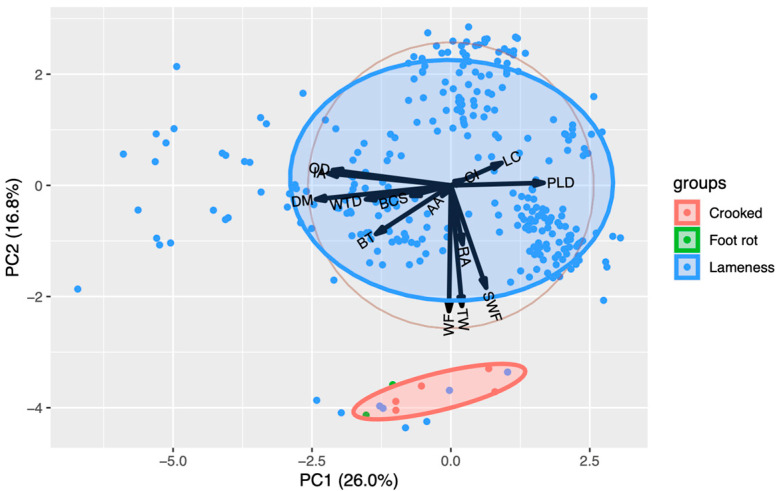
Principal component analysis for legs diseases (LDs) in cows from two farms in northern Mexico. PC1 = Principal component 1; PC2 = Principal component 2, DM = Days in milk, PLD = Last production in 24 h, IA = Breeding days, OD = Open days CI = Calving interval, BCS = Body condition score, BT = Pregnancy time, RA = Ruminant movement average last 24 h, AA = Activity Average in last 24 h, WF = Distance from the edge of the wall to the trap, WTD = Height of the feed crust, and SWF = Height of manure crust adhered to the wall.

The distinction between LDs and the variables with the highest values associated with this group is shown in Figure 4. Results for WF, SWF, and TW were found to be 0.579718, 0.3371, and 0.50856, respectively.

#### 3.2.4. Principal Component Analysis for Dairy Farms

Following the identification of variables associated with the LTDA, another principal component analysis was conducted to ascertain whether the variables exhibiting this association are related to the dairy farms, since the study was conducted on two dairy farms (G60 and G100). The sum of the principal components (PC1 + PC2) is equal to 43.5% (PC1 22.6%, and PC2 20.9%) (Table 4).

**Figure 5:**
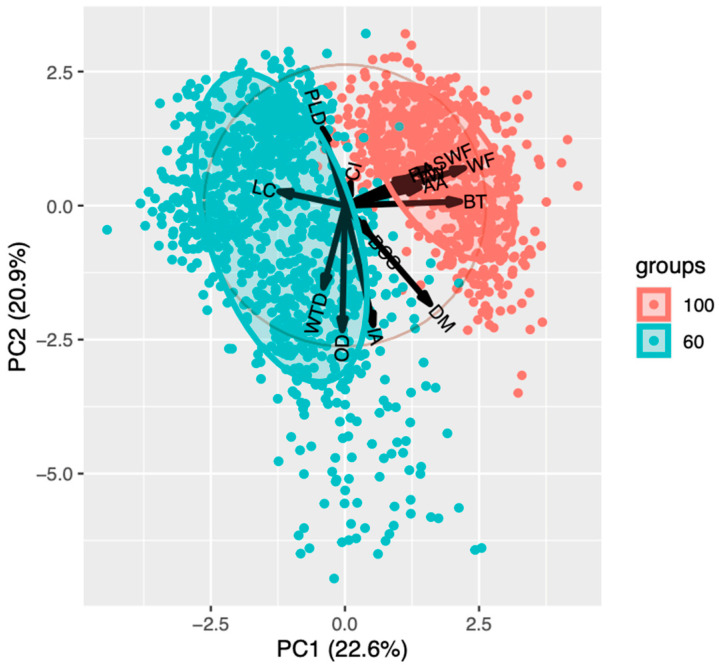
Principal component analysis for dairy farm in cows from two farms in northern Mexico. PC1 = Principal component 1; PC2 = Principal component 2, DM = Days in milk, PLD = Last production in 24 h, IA = Breeding days, OD = Open days CI = Calving interval, BCS = Body condition score, BT = Pregnancy time, RA = Ruminant movement average last 24 h, AA = Activity Average in last 24 h, WF = Distance from the edge of the wall to the trap, WTD = Height of the feed crust, and SWF = Height of manure crust adhered to the wall.

The results demonstrated that the variables associated with the occurrence of crooked cows are related to dairy farm G100 (Figure 5). The analysis demonstrates that the variables with the greatest weight are those pertaining to the total width of the feed bunk. These variables are the manure crust adhering to the feeder wall (WF) and the distance from the edge of the wall to the feeder trap (SWF), which exhibited the following values: WF (0.631) and SWF (0.4955). Another significant associated variable was breeding time (BT), which exhibited a value of 0.4767.

#### 3.2.5. Principal Component Analysis for Pens

Another analysis was developed to investigate whether there is a relationship between these variables and specific pens. The results of the principal component analysis (PCA) indicated that pens 308, 302, and 306 are associated with these specific variables. Principal component analysis was performed on the data set derived from the G100 barn. The sum of the principal components (PC1 + PC2) yielded a value of 43.5%, with PC1 accounting for 22.6% and PC2 for 20.9% (Table 4).

**Figure 6:**
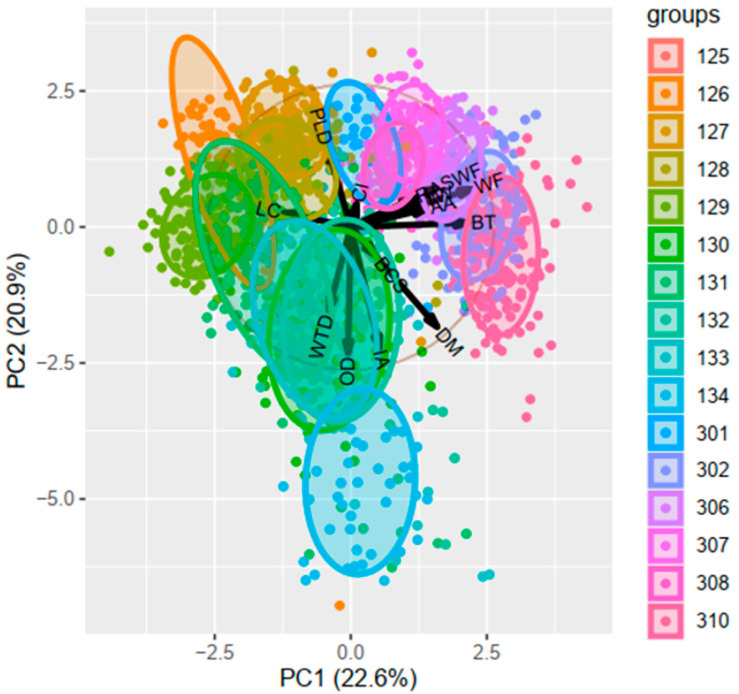
Principal component analysis for corrals/pens of G100 in cows from northern Mexico. PC1 = Principal component 1; PC2 = Principal component 2, DM = Days in milk, PLD = Last production in 24 h, IA = Breeding days, OD = Open days CI = Calving interval, BCS = Body condition score, BT = Pregnancy time, RA = Ruminant movement average last 24 h, AA = Activity Average in last 24 h, WF = Distance from the edge of the wall to the trap, WTD = Height of the feed crust, and SWF = Height of manure crust adhered to the wall.

The analysis shows that the variables with the highest weight were SWF and WF which interacts mainly in pens 308, 302, and 306 (Figure 6). In all barns, the measurements of the wall show a WF value of 0.20 m. The only noticeable difference between these three barns is the presence and height of the manure crust adhering to the wall. The manure crust height for corrals 308, 302, and 306 was 0.24 m, 0.40 m, and 0.30 m, respectively. Furthermore, BT is identified as a significant variable in the analysis. The variable values are as follows: The WF variable has a value of 0.631, the SWF variable has a value of 0.495, and the BT variable has a value of 0.4767.

**Figure 7:**
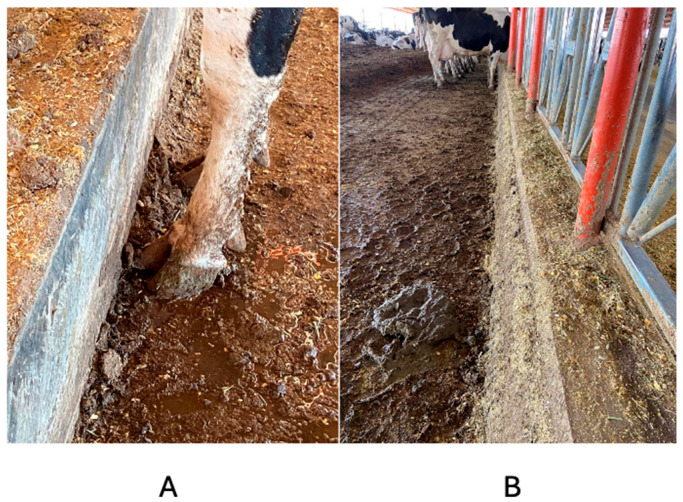
Feeder bunk without crust (**A**) and barn feeder with crust (**B**) of the G100 dairy farm in north-central Mexico.

**Figure 8:**
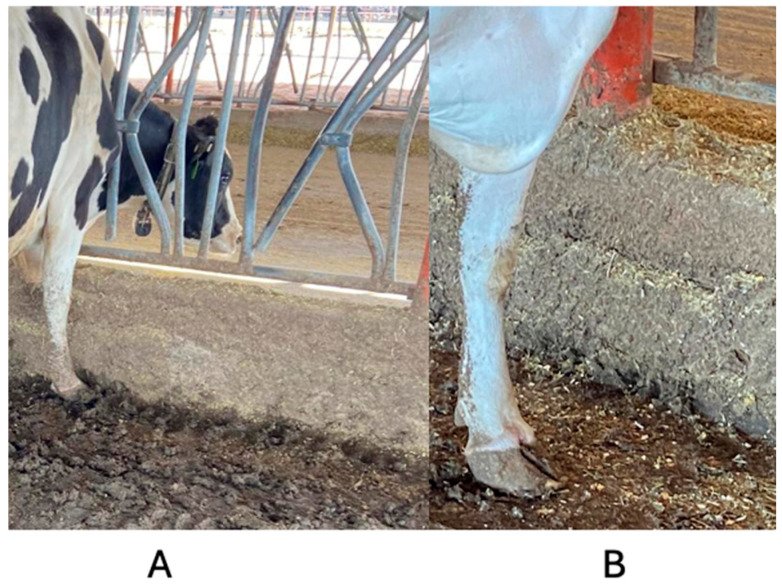
Non-crusted feeder bunk (**A**) and crusted feeder bunk (**B**) of the G60 dairy farm in north-central Mexico.

**Figure 9:**
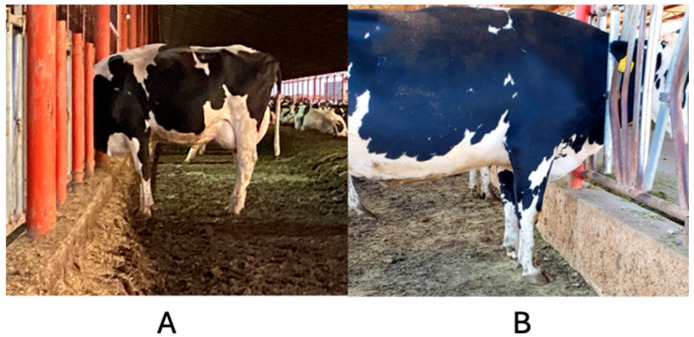
Non-crusted feeder bunk (**A**) and crusted feeder bunk (**B**) of the G60 dairy farm in north-central Mexico.

Figure 7, Figure 8 and Figure 9 illustrate the realistic physical conditions of the G60 and G100 feeder bunks, as well as the manure crusts attached to them and the behavior of the cows in accessing the feed. Figure 7A depicts a G100 feeder bunk, which has been designed in a manner that is more conducive to promoting feed consumption by cows. This is due to the fact that the feeder bunk does not induce the cows to twist their forelimbs, which can predispose them to LTDA. Figure 7B depicts a G100 trough that has accumulated crust, resulting in an increase in width. This impedes the cattle’s ability to access the feed adequately.

Figure 8 illustrates the configuration of feeder bunks on the G60 farm. The initial image (A) depicts a feeder devoid of a crust, whereas the subsequent image (B) illustrates a feeder with a crust. As can be observed, there is a notable difference in the accumulation of crust between the G100 and G60 samples.

Figure 9 illustrates the behavior of cows consuming feed in a G100 pen (A) and in a G60 pen (B). The first cow displays an opening of the limbs, while the second cow does not exhibit this additional effort.

## 4. Discussion

Our results show that the design of the feed bunk on dairy farms, and more specifically the height of the feed curb, affect the health of the front legs, increasing the prevalence of leg torsion and distal asymmetry (LTDA) and leg diseases (LDs) according to feed bunk weight augments. The overall prevalence of LTDA found in this study was 27.6%, with 31.4% in G60 and 22.4% in G100. Solano-López et al. [10] reported a prevalence of limb torsion of 35% in cows with five lactations and 36.2% for cows with six lactations. The bigger prevalence reported by Solano-López et al. could be explained because their study was conducted in Canada, where the cows under investigation remained on slippery floors, in contrast to dairy cows from the Comarca Lagunera region of north-central Mexico, which spent a significant portion of the day directly on soft pen surfaces.

The PCA results indicate that there is no statistically significant association between the variables included in the study and LTDA. Nevertheless, a statistically significant correlation was identified between the variables and LDs. In this case, the variables with the highest weights were WF, SWF, and TW, with values of 0.579718, 0.3371, and 0.50856, respectively. It is likely that the lack of an association in LTDA is a consequence of the failure to include other variables with explanatory capacity, particularly the physical conformation of the cows, given that the cows in the barns included in the study originate from disparate genetic lines. It is widely accepted that the limbs of cattle are symmetrical, however, the available data on this subject are limited [8,25] and few studies have investigated geometric morphometry [14,15], which is inheritable.

The elevated prevalence of LTDA in G60 can be attributed to the augmented dimensions of the feed bunk in this farm (high and wide) in comparison to those in G100, as substantiated by the findings. In this regard, Gündemir et al. [13] conducted an analysis of the phalanges of the hind and forelimbs of Holstein cows obtained from slaughterhouses in the Istanbul region. Morphometric measurements were taken from 144 digital bones, and the researchers concluded that forelimb asymmetry may be related to the fact that the cows were raised and kept on concrete floors. Furthermore, the disparate distribution of stress on the cattle’s legs has been linked to the anatomical positioning of the hooves during feeding in the pen [26]. This is also related to the dimensions of the feed bunk, which restrict the quantity of food that cows can consume at one time during feeding. It seems reasonable to suggest that the higher prevalence in G60 is attributable to the variation in feed bunk height and width across pens. When cows transition between pens based on days in milk, they must adapt to the disparate feed bunk dimensions. In contrast, in G100, all feed bunk dimensions are consistent across pens. In contrast, the feed fences in G60 are taller and wider, which may contribute to increased postural stress for cows on this farm. Moreover, the change of pens is associated with the reproductive condition of the cows (pregnant or not), which may also predispose to the presence of LTDA and LDs. This is because the PCA asserts that breeding time predisposes to these diseases, particularly in pregnant cows [10].

A number of factors have been identified as affecting hoof health, including genetics, diet, infectious agents, hygiene, housing systems, animal behavior, and management practices [27]. In this regard, painful conditions affecting the musculoskeletal system of the cow prompt the animal to alter its gait and posture in order to reduce discomfort. Such changes are evidenced by alterations in movement, gait, or posture [28]. Furthermore, foreleg and hind leg disorders resulting in lameness are more prevalent in higher-yielding management system [29,30]. Furthermore, the ongoing process of selecting and crossbreeding dairy cattle for production and reproductive traits has resulted in the unintended consequence of the neglect of other essential characteristics, such as the structure of the limbs [31]. Consequently, further research is required to ascertain the mechanisms by which the prevalence of distal asymmetry and foreleg rotation can be reduced, thus extending the productive life of cows and improving animal welfare [28].

Lameness represents a significant concern within the dairy industry, given its associated costs and implications for animal welfare [32]. The estimated cost of clinical lameness in dairy cattle is approximately $500 per case [33], which translates into significant economic losses in intensive production systems and concerns regarding animal welfare. In this context, the term “costs” is understood to encompass not only financial losses but also losses pertaining to animal health and welfare [29].

Visual locomotion scoring has been a commonly utilized method for assessing the quality of cow movement. However, it has been demonstrated to be an unreliable and relatively subjective method of locomotion assessment when compared to automated alternatives [26]. The application of kinematic, kinetic, and accelerometric technologies enables the acquisition of more detailed outcome measurements than those obtained through visual scoring [34], which must be evaluated to qualify the possibility of their use in the farms with LTDA problems.

Another issue pertaining to the locomotion of cattle, which is prevalent in highly technical systems and intensive conditions, is directional asymmetry. This phenomenon is evidenced by a significant divergence in direction [35]. This can be attributed to lateralized behavior and biomechanical pressures exerted on a single side of the bovine body [36]. It seems reasonable to suggest that deficiencies in the design of feed bunk structures and their subsequent impact on hoof health (LTDA and LDs) may result in impaired feeding behavior in cows, which in turn could affect the Five Freedoms of Animals as outlined by the Farm Animal Welfare Council [2]. This is because cows experiencing discomfort are unable to express normal behavior, which may manifest as fear and distress. It is evident that foot diseases have a significant impact on the welfare of cattle, leading to alterations in their behavioral patterns. These include changes in their grazing and rumination habits, resting patterns, recumbency, and water intake [37,38]. In this regard, foot health has been identified as the most significant welfare issue in dairy cows, and its monitoring represents the most reliable indicator of welfare in dairy cattle [39].

The presence of LTDA and LDs has the potential to result in the experience of pain, which has the capacity to significantly impair animal welfare. This is due to the aversive nature of pain, the distress associated with the inability to avoid such sensations, and the adverse effects that can negatively impact the animal’s overall quality of life. Furthermore, pain can affect a number of additional functions, including appetite, sleeping patterns, and intestinal function. Consequently, cows afflicted with LTDA or LDs are deprived of the fundamental right to freedom from pain, which is a crucial aspect of animal welfare. The aforementioned conditions have a detrimental impact on a number of factors, including chronic pain, body condition, reproductive capacity, productive potential, and destructiveness [40]. For this reason, stress has been employed as an indicator of diminished well-being, given its capacity to disrupt internal homeostasis through the induction of alterations in autonomic nervous system activity and the hypothalamic–pituitary–adrenocortical axis [41]. The presence of an unevenly sized feeder bunk with a manure crust adhering to it may result in difficulties in accessing feed and gestation, which could potentially lead to stress in the animals. [41]. As a result, the presence of LTDA exerts an influence on a number of aspects pertaining to an animal’s well-being, including its capacity to feed and drink without restriction, its ability to engage in species-specific behaviors, its experience of pain or stress, and its overall health status. This can be attributed to the clinical effects of LTDA.

The present study is limited by a number of factors. Firstly, the exclusion of zoometric variables pertaining to the cattle represents a notable shortcoming in the study. Secondly, the relatively limited number of farms included in the study represents a limitation of the study. Thirdly, the lack of progeny data that could contribute to an understanding of genetic effects represents a further limitation. Additionally, the absence of measurements of chemical indicators of stress, such as cortisol and heat shock proteins, represents a further limitation. Moreover, the absence of progeny data, which could provide insight into genetic effects, represents a significant limitation. The findings of our study suggest a correlation between the configuration of the feed bunk (specifically the dimensions of the feed curb), the cleaning and maintenance of pens, and the prevalence of LTDA in lactating Holstein cows. The dimensions of the feeder bunks in G100 were excessive, which may have contributed to lameness in the legs. This discrepancy between the original concrete dimensions and the accumulation of manure may have been a contributing factor. It is therefore recommended that the G60 pen design, illustrated in Figure 2A, be employed and that there be no variation in pen construction. Moreover, it would be prudent to consider the body size of the dairy animals to be housed when designing feeder bunks. Further research on facility design is recommended, as it can impact both animal welfare and productive life.

## 5. Conclusions

The dimensions of dairy cattle feeder bunks require the cow to expend additional effort to reach the feed when the feeders are of a larger size, due to the increased distance the cow must traverse to access the feed. This results in the limbs being rotated outwards, which can lead to the development of deformed and asymmetrical toes (LTDA).

Moreover, the width of the crust is a contributing factor to the presence of LTDA and LDs. It is thus recommended that dairy farm managers implement enhanced supervision of feed bunks and wall cleaning in order to prevent the accumulation of manure crusts.

The breeding time is associated with the presence of LTDA and LDs, particularly in pregnant cows. It is therefore recommended that greater attention be paid to cows exhibiting this condition, given their heightened predisposition to limb lesions.

If the prevalence of LTDA observed in this study is indicative of the broader population of dairy farms within the complex, it would be prudent to assess the design standards and cleaning procedures of corrals/pens for new dairy facilities. Such an approach would assist in reducing the prevalence of this pathology while simultaneously promoting cow comfort.

## Figures and Tables

**Table 1:** Descriptive statistics of variables in relationship with foreleg torsion distal asymmetry in cows from two farms in northern Mexico. Mean, standard deviation, minimum, and maximum.

Variable	Mean	SD	Min	Max
Lactation number	3.32	1.32	1.00	9.00
Days in milk	130.14	82.58	2.00	571.00
Last production in 24 h	40.38	9.90	0.00	100.65
Breeding services	2.58	1.70	1.00	10.00
Open days	91.34	51.53	2.00	324.00
Calving interval	385.83	71.67	0.00	916.00
Body condition score	3.74	8.71	2.75	325.00
Pregnancy time	61.05	49.22	0.00	215.00
Ruminant movements in last 24 h	275.21	62.83	1.00	598.00
Activity average in last 24 h	247.39	55.04	24.00	833.00
Width of feed bank	0.24	0.05	0.19	0.37
Distance from the edge of the wall to the trap	0.18	0.03	0.13	0.22
Height of the feed bunk	0.32	0.05	0.28	0.46
Width of manure crust adhered to the wall	10.70	4.22	3.00	17.00
Height of manure crust adhered to the wall	21.34	9.05	8.00	40.00
Total width of the feed bunk	10.74	4.15	3.20	16.29

**Table 2:** Categoric variables in relationship with foreleg torsion distal asymmetry in cows from two farms in northern Mexico: *n*, percentage, *χ*^2^, and significance.

	*n*	Percentage	*χ* ^2^	Significance
Foreleg torsion and distal asymmetry				
Negative	3090	72.4	854.36	***
Positive	1180	27.6		
Status				
Abort	2.0	0.0	6081.34	***
Colostrum	354	8.3		
Fresh	324	7.6		
Inseminated	1784	41.8		
Another	101	2.4		
Pregnancy test 1	1162	27.2		
Pregnancy test 2	341	8.0		
Cull	124	2.9		
Empty	78	1.8		
Leg diseases				
Crooked	15	3.2	803.62	***
Foot rot	9	1.9		
Lameness	447	94.0		

Significance level *p* > 0.05, NS; *p* < 0.001 ***.

**Table 3:** Prevalence of limb torsion and distal asymmetry (LTDA) and leg diseases (LDs) in two intensive dairy farms in northern Mexico.

	*n*	G60	G100	*χ* ^2^	Significance
LTDA					
Negative	3090	1706 (68.6)	1384 (77.6)	41.64	***
Positive	1180	780 (31.4)	400 (22.4)		
LDs					
Negative	3799	2046 (82.30)	1753 (98.26)	269.64	***
Positive	471	440 (17.60)	31 (1.74)		

Significance level *p* > 0.05, NS; *p* < 0.001 ***.

**Table 4:** Principal component analysis for foreleg torsion and distal asymmetry (LTDA) in cows from two farms in northern Mexico.

	PC1	PC2
Lactation	−0.2621	0.0584
Days in milk	0.3400	−0.4081
Last production to last 24 h	−0.0895	0.3188
Breeding services	0.1135	−0.4949
Open days	−0.0106	−0.5165
Calving interval	0.0231	0.0947
Body condition score	0.0969	−0.1176
Pregnancy time (BT)	0.4601	0.0166
Ruminant movement average last 24 h	0.2474	0.1162
Activity average 24 h	0.2988	0.0746
Width of the feed bunk wall (WF)	0.4755	0.1555
Distance from the edge of the wall to the trap (WTD)	−0.0865	−0.3400
Width of the manure crust adhered to the wall (SWF)	0.3373	0.1582
Total width (TW) [WTD + SWF]	0.2743	0.0989

PC1 = Principal component 1; PC2 = Principal component 2.

**Table 5:** Principal component analysis for leg diseases (LDs) in cows from two farms in northern Mexico.

	PC1	PC2
Lactation	0.1859	0.1025
Days in milk	−0.4942	−0.0635
Last production to last 24 h	0.3396	0.0115
Breeding services	−0.4499	0.0543
Open days	−0.4323	0.0698
Calving interval	0.0472	0.0263
Body condition score	−0.1450	−0.0479
Pregnancy time (BT)	−0.2745	−0.2219
Ruminant movement average last 24 h	0.0431	−0.2680
Activity average 24 h	−0.0317	−0.0483
Width of the feed bunk wall (WF)	−0.0069	−0.5728
Distance from the edge of the wall to the trap (WTD)	−0.3110	−0.0637
Width of the manure crust adhered to the wall (SWF)	0.1292	−0.4663
Total width (TW) [WTD + SWF]	0.0405	−0.5491

PC1 = Principal component 1; PC2 = Principal component 2.

## Data Availability

The datasets used along with this research could be available from the corresponding author on reasonable request.
